# Widespread Disruptions of White Matter in Familial Multiple Sclerosis: DTI and NODDI Study

**DOI:** 10.3389/fneur.2021.678245

**Published:** 2021-08-16

**Authors:** Zeinab Gharaylou, Mohammad Ali Sahraian, Mahmoudreza Hadjighassem, Mohsen Kohanpour, Rozita Doosti, Shima Nahardani, Abdorreza Naser Moghadasi

**Affiliations:** ^1^Multiple Sclerosis Research Center, Neuroscience Institute, Tehran University of Medical Sciences, Tehran, Iran; ^2^Brain and Spinal Cord Injury Research Center, Neuroscience Institute, Tehran University of Medical Sciences, Tehran, Iran; ^3^Neuroimaging and Analysis Group (NIAG), Research Center for Molecular and Cellular Imaging, Imam Khomeini Hospital, Tehran University of Medical Sciences, Tehran, Iran

**Keywords:** familial multiple sclerosis, DTI, NODDI, TRACULA, brain mapping

## Abstract

Diffusion tensor imaging (DTI) is a noninvasive, quantitative MRI technique that measures white matter (WM) integrity. Many brain dimensions are heritable, including white matter integrity measured with DTI. Family studies are valuable to provide insights into the interactive effects of non-environmental factors on multiple sclerosis (MS). To examine the contribution of familial factors to the diffusion signals across WM microstructure, we performed DTI and calculated neurite orientation dispersion plus density imaging (NODDI) diffusion parameters in two patient groups comprising familial and sporadic forms of multiple sclerosis and their unaffected relatives. We divided 111 subjects (49 men and 62 women: age range 19–60) into three groups conforming to their MS history. The familial MS group included 30 participants (patients; *n* = 16, healthy relatives; *n* = 14). The sporadic group included 41 participants (patients; *n* = 10, healthy relatives; *n* = 31). Forty age-matched subjects with no history of MS in their families were defined as the control group. To study white matter integrity, two methods were employed: one for calculating the mean of DTI, FA, and MD parameters on 18 tracts using Tracts Constrained by Underlying Anatomy (TRACULA) and the other for whole brain voxel-based analysis using tract-based spatial statistics (TBSS) on NDI and ODI parameters derived from NODDI and DTI parameters. Voxel-based analysis showed considerable changes in FA, MD, NDI, and ODI in the familial group when compared with the control group, reflecting widespread impairment of white matter in this group. The analysis of 18 tracts with TRACULA revealed increased MD and FA reduction in more tracts (left and right ILF, UNC, and SLFT, forceps major and minor) in familial MS patients vs. the control group. There were no significant differences between the patient groups. We found no consequential changes in healthy relatives of both patient groups in voxel-based and tract analyses. Considering the multifactorial etiology of MS, familial studies are of great importance to clarify the effects of certain predisposing factors on demyelinating brain pathology.

## Introduction

Multiple sclerosis (MS) is a chronic degenerative disease of the entire neuroaxis that originates from autoimmunity processes and presents diverse clinical manifestations. The multi-factorial pathophysiology of MS has its roots in complicated co-operation between hereditary and non-hereditary (intra-familial and non-intrafamilial) influences in different ways and proportions; many dimensions are still not elucidated (1, 2). Axonal loss is accepted as the final common phenomenon of irreparable and heterogeneous neurological disabilities in MS. Inflammatory demyelination is prominently underpinned by axonal transection and subsequent degeneration. Other contributors, including axonal damage of permanently demyelinated axons and slow axonal burning at the lesion edge, might further result in tissue damage, particularly in the progressive levels of the disease (3, 4).

Compared to all settings, family studies are valuable to provide insights into the interactive impacts of the environmental and non-environmental modules on MS. More recent studies in Canada, the United States, and Northern Europe have established that the risk for MS in first-degree relatives of affected individuals is 20–40 times higher, 300 times higher in monozygotic twins (5). Epidemiological and clinical studies of MS estimate familial aggregation of the disease in up to 20% of cases. In families with parent-child concordance, a higher mother-to-child than father-to-child transmission is observable, indicating a maternal constituent of susceptibility. Many brain dimensions are highly heritable, including whole brain volume (6), regional gray and white matter volumes (7), cortical thickness (8, 9), and white matter integrity measured with DTI (10–12) regarding healthy or disordered states (12, 13). In functional magnetic resonance imaging (fMRI), genomic components account for around 80% of the total diversity in the BOLD signal during working memory-associated tasks (14–16). Measures of default-mode activity observed with resting-state fMRI are also highly heritable (17, 18). In children, white but not gray matter volume heritability grows with increasing age (19), perhaps because white matter volumes continue to rise until the late 40 s (20). Cortical gray matter thickness also becomes more heritable with increasing age in late-maturing areas (21). Some of the same genes could influence the level of integration throughout the white matter as IQ (22); however, no study has investigated the pattern of heritability fluctuations with age. In familial MS patients, diffused brain abnormalities have been detected even at the earliest stages of the disease (23). Magnetization transfer imaging ratio (MTR)-determined tissue integrity revealed more widespread abnormalities in patients with familial MS compared with healthy subjects (24). Familial clustering has been noted for decades (25).

The existing investigations have provided substantial evidence that MS patients' relatives harbor a higher risk for developing this demyelinating disease than others (26). Then, the study of healthy relatives of MS patients might remarkably be significant to clear up the state of the preclinical abnormalities of the brain tracts. The prevalence of radiologically isolated syndrome (RIS) reported in the healthy relatives of MS patients is higher than in other populations (26). MTR studies on asymptomatic relatives of MS patients have shown inconsistent findings, reporting the absence of significant changes in relatives of sporadic MS or lesion brain tissue damage in relatives of both sporadic and familial MS (27, 28). The study showed that specific detectable focal white matter lesions are twice as common in relatives of familial MS patients (27). Meanwhile, various approaches are available for evaluating white matter damage, which is sensitive to different aspects of pathology. Accurate assessment of white matter impairments in MS is valuable in deciding etiologic therapeutic targets and medicinal approaches.

There are two paths to extract information from diffusion data: signal representation (e.g., DTI) and biophysical models (29). One of the most frequently applied methods for analyzing white matter macro- and microstructures is DTI (30). The DTI technique substantially contributes to highlighting the elements of brain microstructural architecture, particularly those that are not visible through conventional sequences. It also provides further insights into the fiber organization, axonal directional coherence, and degree of tract integrity (30). The advanced diffusion model, neurite orientation dispersion and density imaging (NODDI), is of great importance regarding biophysical computations. NODDI was developed to quantify two indices of neurite morphology: the neurite density index (NDI) and the neurite orientation dispersion index (ODI) (31). NODDI has shown more sensitivity than DTI to changes in normal-appearing WM (NAWM) and prognostication of clinical strategies in patients with MS (32). Throughout the current assessment, we have applied two procedural approaches to evaluate the distinctions and deviations in DTI and NODDI indices for addressing more WM integrity alterations. To our knowledge, no study has explored WM integration variabilities in familial and sporadic MS.

Substantial heterogeneity accounts for familial MS prevalence (33), and one of the highest recurrence rates has been reported in Iran (34). Remarkable advances have occurred in our knowledge of white matter (WM), indicating MS pathology through diffusion tensor imaging (DTI) and magnetization transfer imaging.

We conducted the present study to analyze WM microstructure through DTI and NODDI diffusion parameters to demonstrate how familial predisposing factors can affect the integrity of WM of patients with MS. To this aim, we used Tracts Constrained by Underlying Anatomy (TRACULA) (35) of DTI-derived metrics fractional anisotropy (FA) and mean diffusivity (MD) for 18 tracts. Whole-brain voxel-based analysis of FA, MD, and NODDI-derived parameters NDI and ODI was undertaken using tract-based spatial statistic (TBSS) differences between two patient groups, their unaffected relatives, and healthy controls.

## Materials and Methods

**Figure 1:**
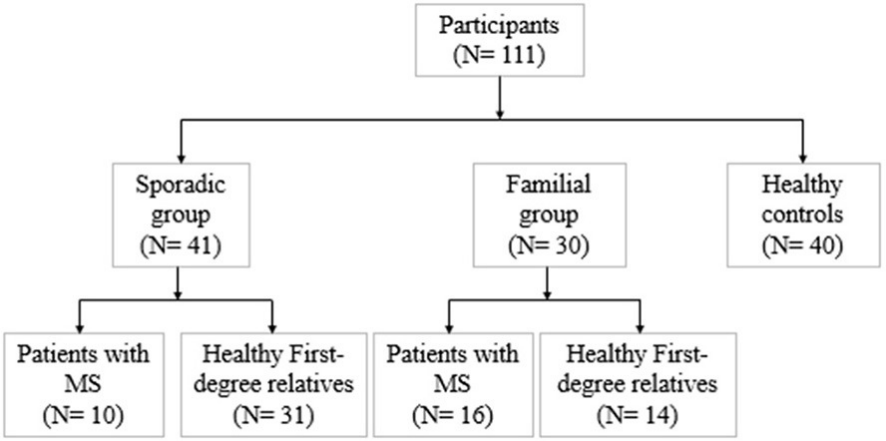
The flow diagram of participants that were allocated to groups.

**Figure 2:**
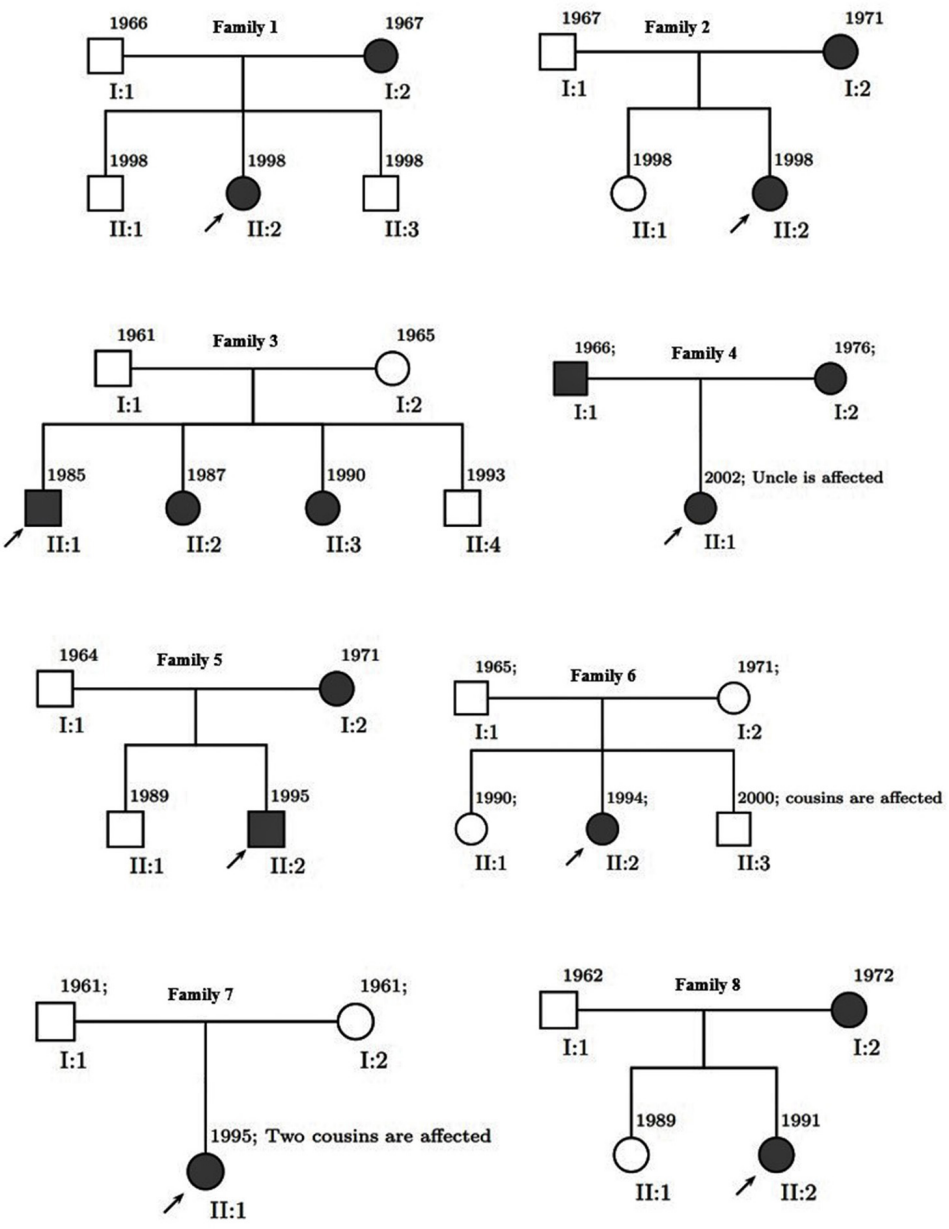
Pedigree of eight families. Men are represented by squares and women by circles. Solid square (male) or circle (female) represent an individual with multiple sclerosis.

We divided 111 subjects (49 men and 62 women: age range 19–60) into three groups conforming to their MS history. The flow diagram of participants allocated to groups is illustrated in Figure 1. Familial MS was defined as families with at least two members with MS from each family. At least one other first- and or second-degree relative had to be confirmed. We represent the family pedigree of eight families with familial MS in Figure 2. The familial MS group included 30 participants (patients; *n* = 16, healthy relatives; *n* = 14). We defined sporadic cases as MS patients with no relatives with MS. The sporadic group included 41 participants (patients; *n* = 10, healthy relatives; *n* = 31). Forty age-matched subjects with no history of MS in their families were defined as the control group. Clinical examination and a comprehensive neuropsychological assessment were performed in the MS Center of University-Affiliated Sina Hospital. MS diagnosis was made according to McDonald's criteria (36), and the patients' clinical status was measured applying the Expanded Disability Status Scale (EDSS) at the entry into the study. Relapsing-remitting cases defined as having relapses and remission but without progression were categorized. Cases with relapses, remission, progression as secondary progression, and finally MS cases with progression, but without relapses and remission, were classified as primary progression. Information about family history and all first- and second-degree family members were obtained by researchers who were blind to patient details through semi-structured interviews with at least one family informant. The participants of both categories (familial and sporadic) were matched regarding clinical status, age, and gender to control the impacts of demographic and clinical characteristics on the findings. Exclusion criteria for patients were any significant neurological or neuropsychiatric disorders that could affect the brain structure. These items included organic brain diseases and previous head trauma resulting in loss of consciousness for more than 5 min, MS patients with depression symptoms, people receiving antidepressant drugs, and contraindications to MRI.

Healthy subjects were recruited from families other than the families with MS cases and evaluated based on the general medical state checklist (head traumas, seizures, and neuropsychological disorders). The selected participants did not report any medication, alcohol, or substance use. Eventually, 40 age and sex-matched subjects consisting of 22 women and 18 men were selected as healthy controls, whose mean age was 39.28 years (range 19–60).

### MRI Data Acquisition

We obtained MRI scans on a 3.0 Tesla Siemens Prisma MRI scanner with a 64-channel head and neck coil. It acquires anatomical images with a high-resolution, T1-weighted MPRAGE (*TR* = 2,250 ms, *TE* = 3.5 ms, flip angle = 7°, FOV = 256 × 256 mm^2^, matrix = 256 × 256 mm^2^, voxel size = 1 × 1 × 0.6 mm^3^, 225 contiguous sagittal slices provided whole-brain coverage). Another image is acquired through 3D-FAST spin echo, T2 fluid-attenuated inversion recovery (FLAIR). This technique is closely related to Fast (Turbo) spin-echo techniques that provide a flexible and robust approach for 3D spin-echo-based imaging with a broad range of clinical applications. We used an isotropic 3D image with voxel size = 0.9 × 0.9 × 0.9 mm^3^, *TR* = 5,000 ms, *TE* = 225 ms, FOV = 256 × 256 mm^2^, matrix = 320 × 320 mm^2^, 176 contiguous sagittal slices provided whole-brain coverage.

The whole-brain DWI data were collected using a single-shot spin-echo EPI sequence with the following parameters: *TR* = 11,000 ms, *TE* = 105 ms, FOV = 256 × 256 mm^2^, matrix size = 128 × 128 mm^2^, flip angle = 90°, voxel size = 2 × 2 × 2 mm^3^, *b* = 700/2,000 with 30/64 directions of diffusion-weighted sensitizing gradients. Extra brain volumes received two non-diffusion weighting (*b* = 0 s/mm^2^) with opposing phase encode directions (anteroposterior and opposite). The total image acquisition time for anatomical and diffusion MRI was not longer than 20 min.

### Image Processing

All diffusion images were concatenated and corrected for eddy current, subject movement, and EPI distortions through Eddy-FSL (https://fsl.fmrib.ox.ac.uk/fsl/fslwiki) (37). First, images were corrected for eddy distortions and motion using an average of the two *b* = 0 s/mm^2^ volumes for each diffusion-weighted shell as a reference. The registered images were skull-stripped using the Brain Extraction Tool DTIFIT (part of FMRIB Software Library, Oxford). The tensor model was applied to corrected DTI data to generate fractional anisotropy (FA) and MD maps from eigenvalues.

The NODDI microstructural model was computed and fitted to the data using the NODDI toolbox (UCL, UK) for Matlab (http://nitrc.org/projects/noddi_tolbox). From this fitting, the derived NODDI indices included the apparent intra-axonal volume fraction vin (NDI), representing the fraction of dendrites and axons; the isotropic volume fraction viso, representing the fraction of free water such as CSF; and the orientation dispersion ODI, a measure of how nonparallel axons are dispersed about a central orientation by assuming a cylindrically symmetric Watson distribution (31).

To perform white matter integrity analysis, we used TRACULA (TRActs Constrained by Underlying Anatomy) for global tractography and tract-based spatial statistics (TBSS) for voxel-wise analyses. For each subject, we used Freesurfer stable version 6.0 (http://surfer.nmr.mgh.harvard.edu) and the recon-all function for automated cortical parcellation as well as subcortical segmentation of T1W images. TRACULA's default tensor fitting and tract reconstruction pipelines using the ball-and-stick model (BEDPOSTX) were applied to the pre-processed data to estimate diffusion probability (part of FMRIB Software Library, Oxford). Subsequently, we used the TRACULA tool (part of Freesurfer version 6.0) for automated reconstruction of 18 major WM tracts including commissural tracts, forceps major (Fmajor) and forceps minor (Fminor), anterior thalamic radiation (ATR), cingulum-angular (infracallosal) bundle (CAB), cingulum-cingulate (supracallosal) bundle (CCG), corticospinal tract (CST), inferior longitudinal fasciculus (ILF), superior longitudinal fasciculus temporal endings (SLFT) parietal endings (SLFP), and uncinate fasciculus (UNC) for each hemisphere. We analyzed the average FA and MD for all tracts.

A TBSS approach was performed to investigate shifts in diffusivity parameters along WM tracts. Once FA maps were measured for all subjects applying the FMRIB diffusion toolbox, FA data from each participant were further processed and analyzed employing the TBSS tool available in FSL. So the TBSS procedure was done. The nonlinear registration algorithm in FSL was used for the normalization of FA images to the standard FMRIB58 FA template. To create the mean FA map, the normalized FA images were averaged. The mean FA map fed into the tract skeleton generation. The skeleton of the tract was a single line (or surface) running down the center of this tract. For FA skeleton generation, a threshold of 0.2 was used to exclude voxels that were primarily gray matter or cerebrospinal fluid. Following FA skeleton generation, an individual subject's FA was projected onto the FA skeleton. We used the nonparametric permutation method in FSL (FSL randomize procedure) to test FA differences between the groups. The threshold-free cluster enhancement at *P* < 0.05 (5,000 permutations) was fully corrected for multiple comparisons using the investigation of WM abnormalities. The exact tract-based analysis method was applied to the MD, NDI, and ODI images.

### Lesion and Volume Evaluation

We applied the Lesion Segmentation Tool (LST) version 3.0.0 which is an open-source toolbox (https://www.statistical-modeling.de/lst.html) for SPM12. LST has been developed for MS lesions segmentation. The lesion prediction algorithm (LPA) has been used for segmenting T2-hyperintense lesions from FLAIR images (38). For the LPA, this algorithm consists of a binary classifier in the form of a logistic regression model trained on the data of 53 MS patients with severe lesion patterns. The covariates used a lesion belief map as for the lesion growth algorithm (39) as well as a spatial covariate that takes into account voxel specific changes in lesion probability. Parameters of this model were employed to segment lesions in new images by providing an estimate for the lesion probability for each voxel. The lesion probability threshold was set to the default value (0.65) (40).

FreeSurfer v6.0 (http://surfer.nmr.mgh.harvard.edu/) was applied for automated segmentation of the T1-weighted images by recon-all command. The file named aseg.stats was created inside the directory “/stats” and during recon-all processing, this file contains the summary of all volumes of the segmented image. For this study, total gray matter volume and total cerebral white matter volume were used.

### Quality Assessment of DTI Data

Considering there is a difference in the signal-to-noise ratio (SNR) in diffusion images, we fulfilled our counting through TRACULA, based on the mean of the signal intensity of whole-brain images. As the SNR in a selected anatomical location in diffusion-weighted imaging can depend on the direction, the SNR in the b0 image is typically reported (41). We calculated SNR for each diffusion-weighted image in gradient direction 0 through DIPY software (v. 1.4.1). We used voxels from the corpus callosum, which have the characteristic of being highly RED in the colored FA map since they are mainly oriented in the left-right direction. First, we computed the tensor model in a brain mask. Next, red-green-blue thresholds were set to (0.6, 1) in the *x*-axis and (0, 0.1) in the *y-* and *z*-axes, respectively. We used all the voxels to estimate the mean signal in this region.

SNR values were averaged for each participant and applied for statistical variability analysis as the differences in head motion between the study groups could induce a false difference in diffusion parameters. The average volume-by-volume translation and rotation, the percentage of signal drop-out, and the averaged drop-out scores with excessive intensity were all computed for each subject. The total motion index (TMI) was obtained from the four motion signs and applied as a nuisance repressor in group analysis.

### Statistics

Statistical analysis of demographic, clinical, and TRACULA results were done using SPSS version 24.0 (SPSS, Chicago, IL) for Windows. Mann-Whitney U (2 samples) tests were used for two-sample comparisons in nonparametric distributions and *t*-tests for parametric cases. We evaluated the difference in sex distribution among groups with the *x*^2^-test. One-way analysis of variance was performed for comparison of age, SNR, and TMI, as well as total white matter and gray matter volume, while an independent non-parametric test was used to compare EDSS, disease duration, lesion number, and volume across the patients with MS.

The mean FA for each subject's tracts calculated by TRACULA based on the probabilistic fibers were analyzed using the general linear model's procedure. Analyses were multivariate for 18 tracts, with values from the left and right hemispheres entered as dependent variables. Sex and group were entered as fixed factors, while age, SNR, and TMI were entered as covariates. The results were Bonferroni-corrected for comparison across tracts. The Spearman rank-order correlation was set to examine the relationship between mean FA and MD for each significant tract and EDSS as well as disease duration. To correct for multiple comparisons, the Benjamini-Hochberg false discovery rate (FDR) procedure was applied, and, after this correction, the level of statistical significance was set at *p* < 0.05.

For voxel-wise analyses (TBSS), the groups were compared using general linear models, covarying age and gender. We estimated the group differences in the white matter measures using an unpaired *t*-test with a nonparametric permutation method (5,000 permutations). The statistical maps threshold was set at *P* < 0.01 using a threshold-free cluster enhancement (TFCE) method with family-wise error (FWE) correction for multiple comparisons. DTI and NODDI maps (FA, MD, ODI, and NDI) that were abnormal compared to normal were selected for further correlational analysis within the familial and sporadic MS groups with EDSS and duration of disease. Univariate regression analyses were conducted for each diffusion metric via randomized permutation. The model included EDSS and duration of disease as covariates. The TFCE option was used in the permutation test, which gives cluster-based thresholding for family-wise error correction. As a result, the TFCE *p*-value images obtained were fully corrected for multiple comparisons across space.

## Results

### Quality Assessment

The SNR comparison showed no significant difference between groups: *F*_(2, 111)_ = 2.397; *P* = 0.09 (mean ± SDs were as follow: controls: 3.22 ± 0.43; familial group: 3.05 ± 0.32; sporadic group: 3.05 ± 0.40). The average SNR in the corpus callosum for each diffusion-weighted image in gradient direction b0 is presented in Supplementary Figure 1. The SNR comparison in this direction showed no significant difference between groups.

There were no significant differences either in TMI between groups in two comparison settings (Supplementary Table 1).

### Demographic and Clinical Characteristics

In all, 26 patients had MS, 10 patients were sporadic cases (median age 30 years (IQR 19–40), and 16 patients had familial MS (median age 34 years (IQR 19–53). None of the patients had experienced relapses or corticosteroid therapy during the six months before the study. Patients were on stable disease-modifying treatment or no treatment for at least 3 months. Treatment data are shown in Table 1. There were no significant differences in age (*P* = 0.47), disease duration (*P* = 0.49), and EDSS scores (*P* = 0.36) between the two patients' groups. Table 1 outlines the demographic and clinical data of the participants.

**Table 1:** Demographic and clinical variables according to multiple sclerosis group.

	**Groups**			**Stat**
**Total information**	**Controls**	**MS familial**	**MS sporadic**	***p*-value**
Number of subjects	40 (36.09%)	30 (27%)	41 (36.9%)	-
Sex (female/male)	22/18	16/14	24/17	0.95
Handedness (right/left)	38/2	26/4	40/1	0.07
Age (years)	35 (19–60)	35 (19–60)	37 (19–60)	0.94
**PATIENT INFORMATION**				
Number of patients	-	16/30	10/41	
Age (years)		34 (19–53)	30 (19–40)	0.47
Disease duration (mean) (years)	-	6.5 (1–18)	7 (1–12)	0.49
EDSS score (range)	-	2 (1.5–3)	1.75 (0–6.5)	0.36
Lesion volume (cm^3^)	-	2.44 (0.344–55.82)	1.410 (0.111–18.85)	0.69
Lesion number	-	14.50 (0–30)	17 (2–23)	0.80
Total WM volume (cm^3^)	445.5 (361.3–571.5)	426.5 (320.9–563)	412.5 (311.7–566.6)	0.0001
Total GM volume (cm^3^)	636.9 (531–676.8)	625.3 (505.8–724.8)	598.3 (504.1–796.6)	0.09
MS treatments				
Ritoxan		3/16	4/10	
Synovox		5/16	2/10	
Cinnomer		5/16	4/10	
Betaferon		1/16		
Resigen		2/16		

*The conducted statistical tests were Chi-squared tests of independence for sex and handedness; one-way ANOVA for age; and nonparametric unpaired Mann-Whitney test for all remaining clinical variables. Variables presented with median (IQR)*.

Median and IQR of lesion volume and lesion number, as well as total white matter and gray matter volume are provided in Table 1. There were significant differences in total white matter of both patient groups and healthy subjects (*p* = 0.0001). There were no differences in total gray matter volume between patient groups and controls (*p* = 0.09). We found no significant difference in lesion numbers and volume in the patient groups.

### Voxel-Wise DTI Differences Across Groups

**Figure 3:**
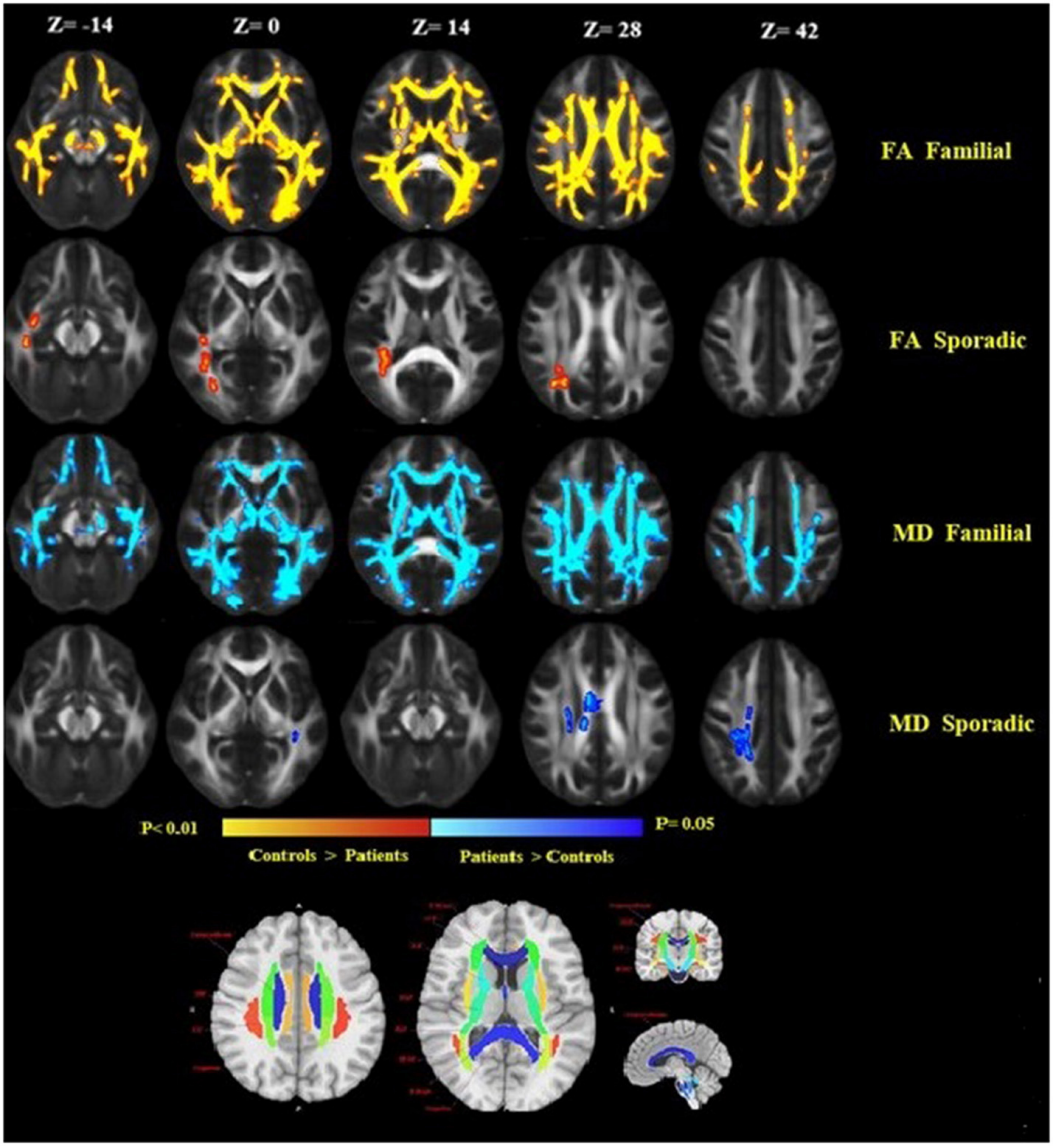
Widespread alterations in DTI-derived fractional anisotropy (FA) and mean diffusivity (MD) found in patients as demonstrated by TBSS. FA and MD show regional differences in patients as compared to healthy controls. FA is lower throughout the white matter in patients as compared to healthy controls, while MD increased in patients compared to controls. Significant changes in FA and MD were found in the familial group, reflecting widespread disruption of white matter. Images are displayed in radiological convention with a left hemisphere on the right side. DTI location changes in FA and MD are presented based on an anatomical atlas (JHU White Matter Tractography Atlas). Fmajor, forceps major; Fminor, forceps minor; ATR, anterior thalamic radiation; CST, corticospinal tract; ILF, inferior longitudinal fasciculus; SLF, superior longitudinal fasciculus; IFOF, inferior-fronto-occipital fasciculus.

Comparison between familial MS patients and the control group showed widespread diffusion-related changes (diminished FA and increased MD). Familial MS patients had significant FA changes in regions including left and right ATR, CST, inferior fronto-occipital fasciculus, SLFT, ILF, right cingulum, body, and splenium of the corpus callosum. The MD index increased in the right and left CST, ATR, SLFT, inferior fronto-occipital fasciculus, cingulum, right ILF, and body of the corpus callosum (Figure 3). In the sporadic group compared with controls, FA showed changes in the right ILF and inferior fronto-occipital fasciculus. Also, MD increased in the body of the corpus callosum as well as right superior corona radiata. There were no significant differences between patient groups. In comparisons between healthy relatives of both MS patient groups with the control group, no significant differences were observed.

### Voxel-Wise NODDI Differences Across Groups

**Figure 4:**
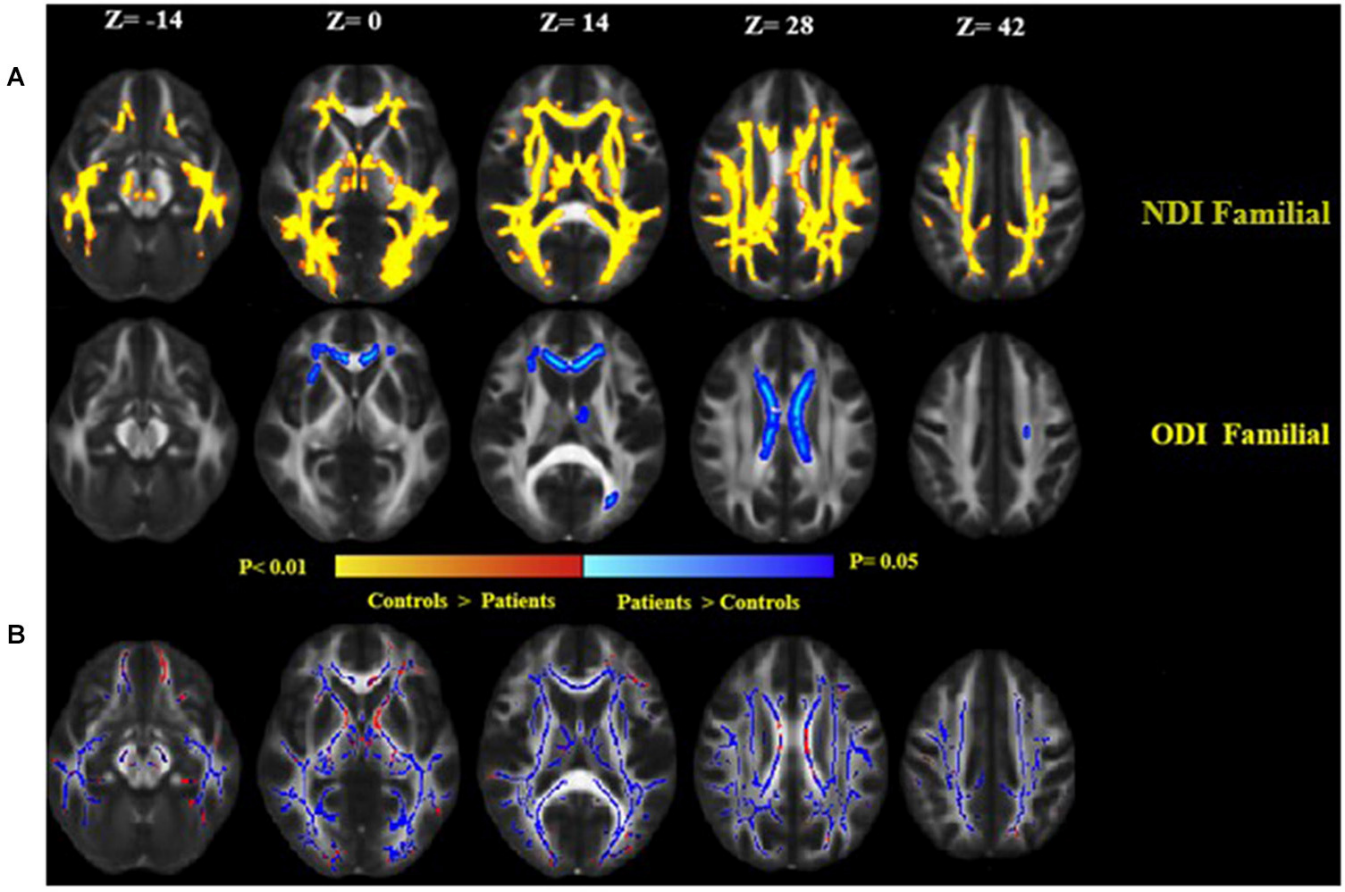
**(A)** Widespread alterations in NODDI parameters [neurite density index (NDI) and neurite orientation dispersion index (ODI)] found in patients as demonstrated by TBSS. NDI and ODI show regional differences in patients as compared to healthy controls. NDI was lower throughout the central white matter in patients as compared to healthy controls, while ODI increased in patients than controls. Significant changes in NDI and ODI reflect widespread disruption of white matter in the familial group. **(B)** Clusters of voxels with significantly decreased NDI in blue are overlaid on the FA. FA was lower in the white matter in familial MS patients as compared to healthy controls (red). Images are displayed in radiological convention with a left hemisphere on the right side.

Familial MS patients compared with healthy controls showed a lower NDI in widespread WM brain regions consisting of the right and left corticospinal tract, SLFT, ILF, inferior front-occipital fasciculus, left anterior thalamic radiation, and body of corpus callosum. ODI increased in the body of the corpus callosum (Figure 4A). To investigate the correspondence between FA changes and regions with a diminished NDI, we superimposed the NDI map onto the FA map (Figure 4B). There were no significant differences between the patient groups. There were no significant differences either in the healthy relatives of both MS patient groups in NODDI indices.

### Tract Analysis Differences Across Groups

**Figure 5:**
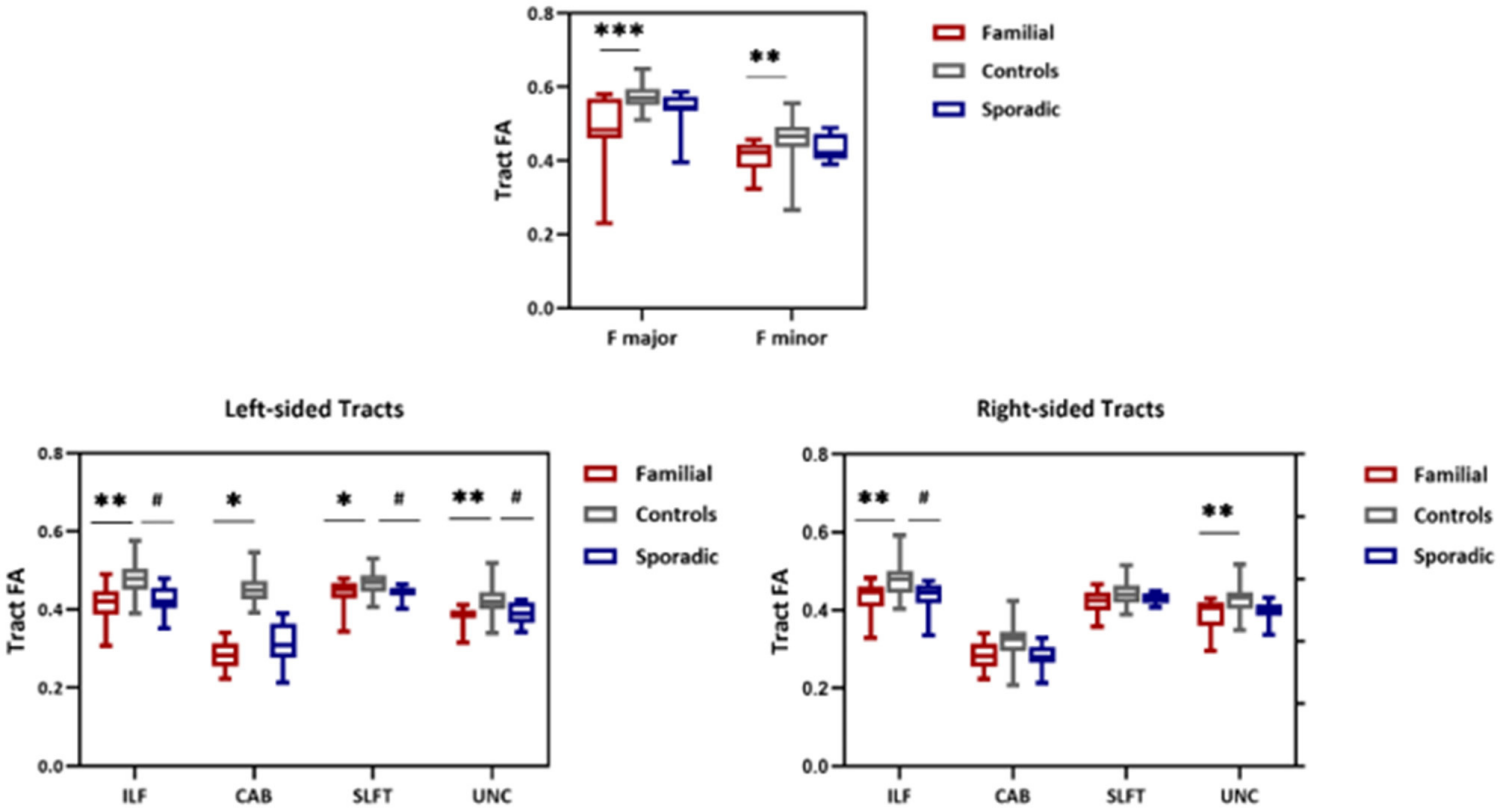
FA values from TRACULA tracts Fmajor, Fminor, ILF, SLFT, UNC, and CAB. The plot shows mean FA left and right tracts and Fminor and major of patients with familial MS (red bars), controls (gray bars), and patients with sporadic MS (blue bars). Asterisks and number signs show significantly decreased FA values for patients when compared to controls. *p < 0.05; **p < 0.01; #*p* < 0.05.

**Figure 6:**
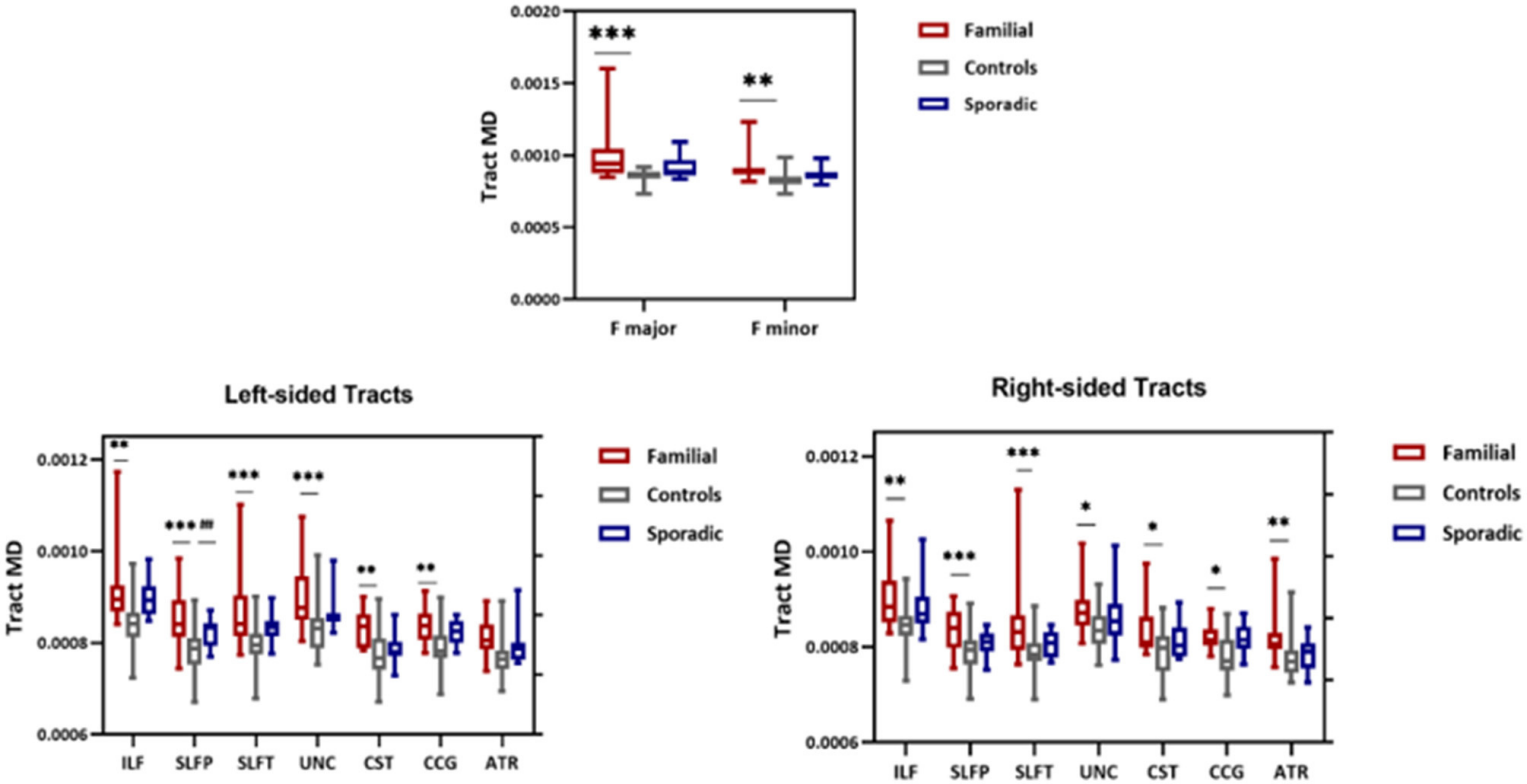
MD values from TRACULA tracts Fmajor, Fminor, ILF, SLFT, SLFP, UNC, CST, CCG, and ATR. The plot shows mean MD left and right tracts and Fminor and major of patients with familial MS (red bars), controls (gray bars), and patients with sporadic MS (blue bars). Asterisks and number signs show significantly increased MD values for patients when compared to controls. *p < 0.05; **p < 0.01; ***p < 0.01; ^*##*^*p* < 0.05.

Whole-tract analysis with TRACULA revealed diminished FA in forceps major, minor, right, and left ILF, UNC, left CAB, and SLFT in familial MS cases compared to age and sex-matched controls. However, FA changed in the right and left ILF, left SLFT, and UNC in sporadic MS patients (Figure 5). MD increased in forceps major, minor, right, and left ILF, UNC, SLFT, SLFP, CCG, CST, and left ATR in patients with a family history of MS. MD changed only in the left SLFP tract of sporadic MS patients (Figure 6). There were no significant differences between the patient groups. We found no substantial changes in healthy relatives of both patient groups (Supplementary Table 2).

### Associations Between DTI and NODDI Alterations, EDSS, and Disease Duration

**Figure 7:**
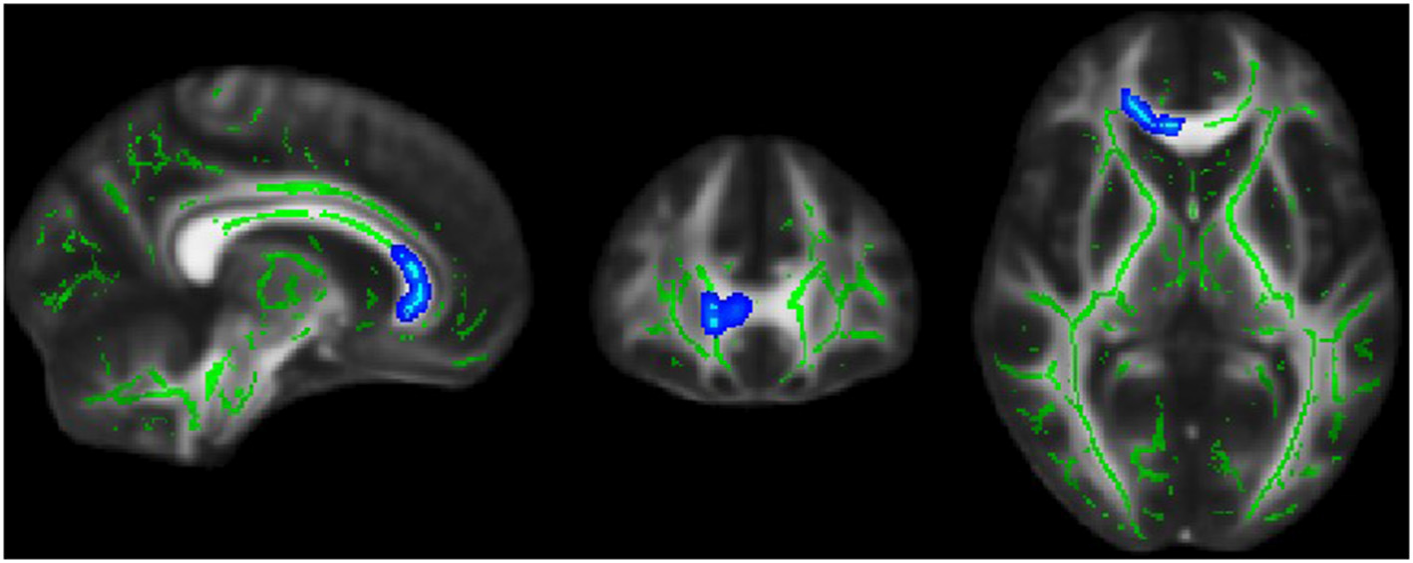
Blue region where white matter neurite orientation dispersion index (ODI) was positively associated with EDSS score in familial MS patients.

Significant correlations were found between ODI and EDSS across familial patients with MS. Figure 7 displays areas of significant correlation. ODI was found to be correlated with EDSS in the corpus callosum (Figure 7). A significant Spearman rank-order correlation was observed between FA, MD, and EDSS and duration of disease settled in the left and right ILF, SLFT, UNC, Fmajor, and Fminor tracts (Table 2). While EDSS and duration of disease increased, MD increased, and FA reduced in these tracts.

**Table 2:** Spearman correlation analysis between FA and MD in tracts that changed significantly in the study with EDSS scores.

			**Left hemisphere**				
	**Variables**	**Pearson correlation**	**ILF**	**SLFT**	**UNC**	**F** ** major**	**F** ** minor**
EDSS	FA	*r*	−0.292^**^	−0.221^*^	−0.270^**^	−0.339^**^	−0.233^*^
		*P*-value	0.04	0.3	0.08	0.01	0.2
	MD	*r*	0.311^**^	0.415^**^	0.384^**^	0.523^**^	0.305^**^
		*P*-value	0.02	0.0001	0.0001	0.0001	0.03
			**Right hemisphere**				
	FA	*r*	−0.211^*^	−0.109	−0.232^*^		
		*P*-value	0.3	1	0.23		
	MD	*r*	0.262^**^	0.400^**^	0.158		
		*P*-value	0.1	0.0001	1		
			**Left hemisphere**				
Duration of disease	FA	*r*	−0.386^**^	−0.330^**^	−0.329^**^	−0.638^**^	−0.276^**^
		*P*-value	0.001	0.01	0.01	0.0001	0.06
	MD	*r*	0.362^**^	0.559^**^	0.480^**^	0.580^**^	0.452^**^
		*P*-value	0.001	0.001	0.001	0.001	0.001
			**Right hemisphere**				
	FA	*r*	−0.332^**^	−0.211^*^	−0.404^**^		
		*P*-value	0.009	0.38	0.0004		
	MD	*r*	0.384^**^	0.569^**^	0.337^**^		
		*P*-value	0.001	0.001	0.001		

* *, correlation is significant at the 0.05 level (two-tailed).*

** *, correlation is significant at the 0.01 level (two-tailed)*.

## Discussion

Differentiating familial MS from sporadic MS is crucial in determining the pathophysiology of MS. In the current study, we have provided the first detailed *in-vivo* characterization of white matter axonal integrity across familial and sporadic MS patients, their healthy relatives, and controls by integrating DTI and NODDI analysis with global tractography analysis TRACULA and a standard voxel-wise group inference technique TBSS. In DTI analysis, more white matter abnormalities were detected in patients with familial MS compared with the control group, while both patient groups showed a smaller total white matter volume. There were no significant differences in lesion volume and number between the patient groups. Using NODDI, we observed a widespread reduction in axonal density (NDI) and increased neurite dispersion (ODI) in familial MS patients. There were no notable differences between patient groups. By considering patients matched for variables that affect the disease, the observed difference might not be apparent because of the clinical characteristics of both patient groups. In sporadic MS and healthy relatives of both patient groups, no significant changes were detected in NODDI and DTI indexes.

Previous epidemiological, genetic, and clinical studies have reported differences between familial and sporadic MS (42). Nevertheless, the cause of these differences has not been identified; it could be the genetic trait or pathogenesis heterogeneity of the disease. Technological advances in research has led to advanced MRI measures, such as MTR, magnetic resonance spectroscopy (MRS), diffusion imaging, and relaxometry techniques which are relatively more specific and sensitive for determining underlying pathology (43). Although MTR is a marker of myelin content in tissues, it is difficult to use it to describe the impact of a particular biophysical phenomenon such as inflammation, edema, axon loss, and demyelination (44). Slight metabolite ratios changed in familial and sporadic MS; it was not correlated with loss of WM integrity (45). However, the existing relevant evidence for comparing familial and sporadic MS was obtained through non-conventional MRI. DTI is a practical approach that can quantify parameters of WM microstructure. These methods may be sensitive to various pathological processes. Under hereditary control, genetic factors affecting total brain volume, regional gray and WM volumes, cortical thickness, and WM integrity are measured with DTI. These studies suggest a relationship between genetic markers and brain white matter damage (46). Postmortem studies showed the correlation of diffusion findings (decreased FA and increased MD) with histopathology in patients with MS (47). Here, we observed that widespread FA declines and MD increases in familial MS patients. Family studies have demonstrated that family factors increase the possibility of a progressive clinical course of MS (42). Further, progressive tissue damage in MS lesions is correlated with progressive brain atrophy, which may reflect demyelinated axons (48). The peak width of skeletonized mean diffusivity (PSMD) is a novel, fully automated MRI metric suggesting a more severe normal-appearing white matter (WM) involvement in MS (49). A significant association was reported between the NLRP1 (NLR family, pyrene domain containing 1) gene and familial MS pathophysiology and neurodegeneration (50). DTI-derived metrics can determine the microstructural changes that further characterize diffuse degeneration across MS patients (51). However, complete studies are required to determine whether these abnormal diffusion indices will develop into MS and whether these variations are correlated with the genetic risk for it.

Several factors reduce FA, such as reduced neurite density as well as increased dispersion of orientation. MD as a parameter is influenced by free space increases under edema, loss of myelin, and axons (52). DTI has limited capabilities to infer specific microstructural changes. To provide valuable insights into the likely nature of these abnormalities, we applied the NODDI analysis method. Advanced biophysical imaging models are used to forecast neurodegeneration and excitability alterations in neuroinflammation (53). NODDI offered a more significant tissue characterization of microstructure abnormalities in the morphology of neuritis. Axonal density (indexed by NDI) is a more specific estimate of density and a more sensitive quantitative indicator of axon pathology than FA (29). Introductory studies have indicated that NDI decreased in lesions and NAWM in MS patients (32, 54). In this study, NDI was reduced in familial MS patients. Further, NDI reduction regions overlapped with those displaying FA reductions. The present results provide considerable insights. Joint NODDI and DTI analyses suggest that the widespread decreased anisotropy is explained by reduced axonal density within white matter pathways, which cannot be interpreted with DTI metrics. Despite overall reductions in axonal density, the dispersion of the neurite structures increases (as indexed by ODI) in the corpus callosum. A recent study has confirmed that NODDI models can also provide substantial heritability estimates in WM and GM (55). Genetic association studies on these heritable diffusion traits are necessary to understand the neurobiology of the underlying diffusion abnormalities.

The strength of this study was applying both TBSS and probabilistic tractography. We evaluated diffusion properties in the WM skeleton and the verified anatomical WM pathways. TBSS is sensitive to localized variations in WM integrity (56), while tractography analysis can calculate the DTI indices to detect changes that diffuse along the length of the tract (57). So, the TBSS might not notice the modifications obtained by tractography (58). Using TRACULA, we found that familial patients had significantly lower FA and higher MD in a set of more tracts. These connectivity measures enhance their potential as indicators of disease progression in MS. To our knowledge, no reports of reduced WM integrity of the tracts in familial and sporadic MS have been published so far.

The frequency of lesions in white matter indistinguishable from those of MS among asymptomatic first-degree relatives ranged from 4 to 10% (27), and the frequency of radiology isolated syndrome among healthy family members was higher in the healthy relatives of patients with MS patients compared with non-familial healthy control subjects (59). A neuroimaging study showed early evidence of the disease in asymptomatic family members whose risk profiles were higher for MS susceptibility (60). In the present study, we evaluated the WM integrity of healthy relatives of both group patients. Although, contrary to our hypothesis, healthy relatives did not exhibit significant variations in DTI and NODDI indexes in both groups. We assessed the healthy relatives of patients with MS with conventional T1–T2 imaging. But, diffusion imaging methods allow for investigating the brain microstructure by measuring water diffusion properties affected by biologic activity. So, advanced imaging biomarkers are more sensitive to MS-specific microstructural changes than conventional imaging. WM signal abnormalities were reported in individuals at vascular disease risk in traditional MRI, so we should interpret these abnormalities.

It reported that familial patients have a more severe and progressive form of the disease. Although there were no differences in disability according to EDSS score between both MS patient groups, our study revealed that in MS patients with family history, the EDSS score correlated with FA, MD, and ODI changes in WM. The present study results indicate that hereditary factors may have a prominent role in neuronal structure damage and neuronal density reduction in patients with MS and possibility of disability progression.

One of the most common causes of degeneration is myelin destruction and inability induced by MS. Many investigations have shown that in relapsing-remitting MS, CIS, and secondary progressive MS, the quantity and volume of lesions are predictive of the development of white matter degeneration (61–64). The present study's findings identified changes in total white matter but not gray matter volume in patients with familial and non-familial MS in comparison with controls. There were no significant variations in lesion volume and quantity between the patient groups. The growing body of evidence supports the view of MS as a disease of WM and GM (65). The mechanisms responsible for the inter-individual variation in the extent of GM and WM pathology are largely unknown. Genetic or environmental factors could influence the core pathologic process and produce identifiable phenotypic variations. Patients with non-vascular etiology of WM lesions like MS can have heterogeneous presentations, which might partially reflect the variety of the clinical disease course and the evolution. While familial MS was associated with more severe T1-lesion volume, there were no clinical status differences between family and sporadic MS patients (66). Whereas, genetic factors can make familial MS patients more inclined to develop the disease than sporadic patients, other factors such as the environmental influence and the subjective (genetic) differences in response to injury seem to be critical for developing a diffuse and perhaps clinically significant development-relevant pathology in MS. Biologic confounders could influence the analysis of GM volumes, including the introduction of disease-modifying therapies, physiologic factors, normal aging, comorbidities, and daily fluctuations in brain volumes (67). The sample size is small and requires validation by encompassing more significant numbers of patients. The fact that directionality of water diffusion changes throughout the WM after adjusting for atrophy suggests that reductions in FA show alterations in white matter not predicted with atrophy. Our findings showed widespread microstructure white matter destruction in the familial MS group. Further, based on considerations, FA and white matter volume are weakly related to the unique characteristics of WM integrity (68, 69).

## Conclusion

We analyzed WM microstructure through diffusion parameters to demonstrate how predisposing genetic factors can influence the integrity of the cerebral white matter of patients with MS. We confirmed differences in brain WM measured by DTI metrics in familial and sporadic MS patients compared to healthy controls; differences in NODDI measures were only observed in familial MS patients. Differences in DTI and NODDI measures between sporadic and familial MS patients remained insignificant. Our failure to find differences in diffusion parameters is not consistent with the studies reviewed in the introduction. The following are possible explanations for the negative results: (1) We implemented methodological approaches to minimize minor sample effects by matching patients and controls in age, sex, and their clinical features: it was not possible to detect group differences in WM because of the lack of statistical power. (2) Although, DTI measurements of white matter microstructure have, in general, high heritability (70), like other brain-related traits, white matter has a complex and highly polygenic genetic architecture (71). However, its genetic underpinnings and relevant biological pathways remain unclear. Based on our current findings, genetic susceptibility may not be sufficient to alter WM tract integrity significantly. Moreover, the sample size is essential to boost genetic power with small effect sizes, explaining our lack of differences. (3) Other influences, including environmental (72), economic (73), lifestyle (74), and genetic predisposition factors, may play independent or combined roles in the WM abnormalities seen in MS. Further research on larger populations is needed and should include genetic data analyses. (4) Gray matter structures, including the cerebral cortex and various deep nuclei, are known to affect MS (67). Our analyses are limited to the WM but can extend to evaluate neurite morphology and potential changes in the GM and include more extensions of the NODDI model to explain anisotropy of the orientation dispersion. Overall, this study provides modest evidence for the usefulness of the familial/sporadic dichotomy as a method to identify and delineate the WM microstructural correlates of genetic factors in MS. Our results await reaffirmation with a more extensive set of MS patients soon.

## Data Availability Statement

The raw data supporting the conclusions of this article will be made available by the authors, without undue reservation.

## Ethics Statement

The ethics committee of Iran University of Medical Sciences reviewed and approved the study protocol (IR.IUMS.REC.1398.863). The patients/participants provided their written informed consent to participate in this study. Written informed consent was obtained from the individual(s) for the publication of any potentially identifiable images or data included in this article.

## Author Contributions

ZG contributed to study design, method definition, data analysis, statistics, and manuscript writing. MS and MH contributed to data interpretation and manuscript editing. MK contributed to study design, MRI acquisition, and manuscript editing. RD and SN recruited the subjects. AM contributed to study design and recruitment, and supervised the study and manuscript editing. All authors contributed to the article and approved the submitted version.

## Conflict of Interest

The authors declare that the research was conducted in the absence of any commercial or financial relationships that could be construed as a potential conflict of interest.

## Publisher's Note

All claims expressed in this article are solely those of the authors and do not necessarily represent those of their affiliated organizations, or those of the publisher, the editors and the reviewers. Any product that may be evaluated in this article, or claim that may be made by its manufacturer, is not guaranteed or endorsed by the publisher.
