# Supplementary material for: Widespread Disruptions of White Matter in Familial Multiple Sclerosis: DTI and NODDI Study
Source: Front Neurol. 2021 Aug 16;12:678245. doi: 10.3389/fneur.2021.678245 (PMC8415561; doi:10.3389/fneur.2021.678245)
Supplement: Supplementary file 1 [file Table_1.DOCX]

**Supplementary Materials**

The head motion parameters described in this study [1]. Total motion index (TMI) was calculated for the i-th subject based on the formula [1] is given by:

where j = 1,…,4 indexes the four motion measures as mentioned above, x_ij_ is the value of the j-th motion measure of the i-th subject, and M_j_, Q_j_, and q_j_ are the median, upper quartile, and lower quartile of the j-th motion measure over all subjects included in a group comparison.

| **Supplementary Table 1.** Head motion parameters for the participants in controls, MS familial and sporadic. | | | | | |
| --- | --- | --- | --- | --- | --- |
|  | **Group** | | | **Stats** | |
| **Head motion parameters** | **Controls** | **MS familial** | **MS sporadic** | **Statistic** | **P value** |
| **Translation (mm)** | 0.48 ± 0.16 | 0.41 ± 0.10 | 0.51 ± 0.24 | F(2,111) = 2.11 | 0.12 |
| **Rotation (degrees)** | 0.003 ± 0.0012 | 0.002 ± 0.0061 | 0.003 ± 0.0024 | F(2,111) = 1.016 | 0.36 |
| **Drop-out percentage** | 0.00 ± 0.00 | 0.00 ± 0.00 | 0.00 ± 0.00 | F(2,111) = 1.102 | 0.33 |
| **Drop-out severity** | 1.00 ± 0.00 | 1.00 ± 0.00 | 1.00 ± 0.00 | F(2,111) = 1.102 | 0.33 |

Translation, average volume-by-volume translation; Rotation, average volume-by-volume rotation angles; Drop-out percentage, percentage of slices with signal drop-out; and Drop-out severity, signal drop-out severity.

**References:**

1. Yendiki, A., et al., *Spurious group differences due to head motion in a diffusion MRI study.* Neuroimage, 2014. **88**: p. 79-90.


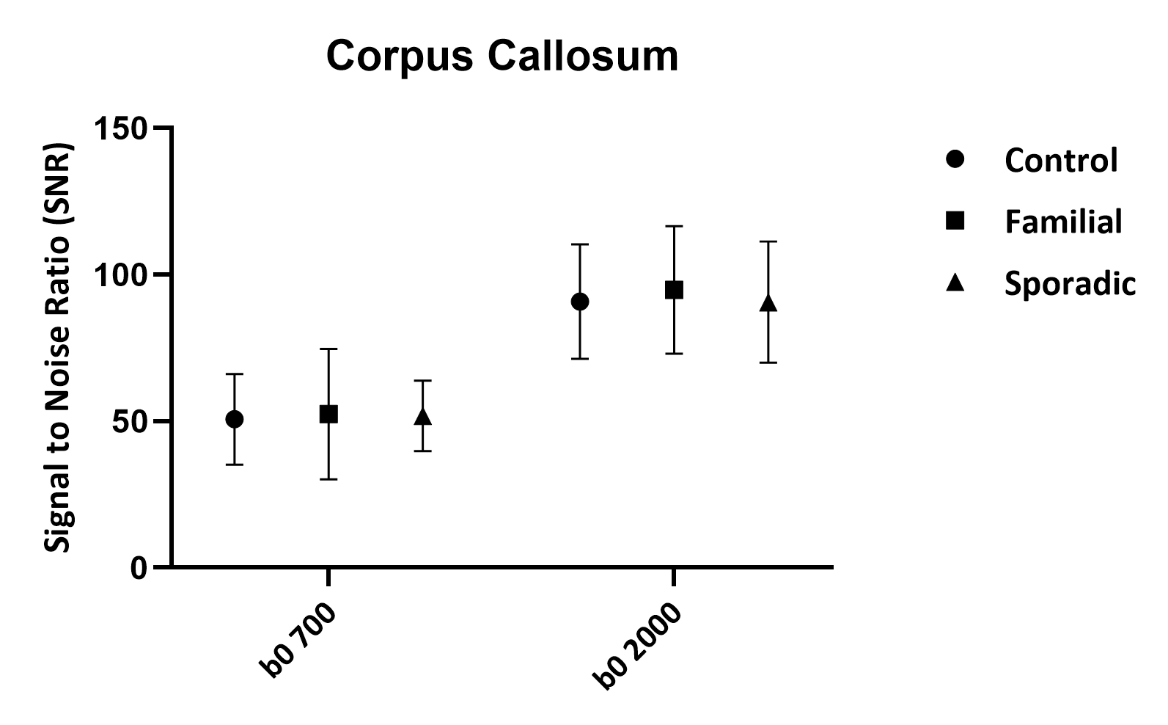


**Supplementary Figure 1: The average SNR in the corpus callosum in gradient direction 0 for b value 700, and b value 2000. Data presented as mean and standard deviation.**

**Supplementary Table 2:** DTI parameters along the entire pathway in healthy relatives of familial and sporadic MS patients and controls.

| **(A)** |  |  | **FA** | **MD e^-3^** |  | **FA** | **MD e^-3^** |
| --- | --- | --- | --- | --- | --- | --- | --- |
| **Hemisphere** | **Tracts** | **Groups** | **Mean (std)** | | **Hemisphere** | **Mean (std)** | |
| **Left** | **ATR** | **control** | **0.44 (0.0330** | **0.76 (0.03)** | **Right** | **0.44 (0.035)** | **0.77 (0.03)** |
|  |  | **Familial relatives** | **0.44 (0.037)** | **0.78 (0.03)** |  | **0.44 (0.052)** | **0.77 (0.05)** |
|  |  | **sporadic relatives** | **0.43 0.029)** | **0.77 (0.03)** |  | **0.44 (0.035)** | **0.76 (0.03)** |
|  | **CAB** | **control** | **0.34 (0.03)** | **0.88 (0.07)** |  | **0.31 (0.043)** | **0.93 (0.1)** |
|  |  | **Familial relatives** | **0.31 (0.064)** | **0.98 (0.032)** |  | **0.33 (0.092)** | **0.94 (0.1)** |
|  |  | **sporadic relatives** | **0.32 (0.044)** | **0.92 (0.07)** |  | **0.33 (0.072)** | **0.9 (0.06)** |
|  | **CST** | **control** | **0.52 (0.06)** | **0.79 (0.04)** |  | **0.52 (0.05)** | **0.77 (0.04)** |
|  |  | **Familial relatives** | **0.53 (0.06)** | **0.79 (0.03)** |  | **0.55 (0.055)** | **0.77 (0.1)** |
|  |  | **sporadic relatives** | **0.52 (0.047)** | **0.8 (0.04)** |  | **0.52 (0.037)** | **0.78 (0.04)** |
|  | **CCG** | **control** | **0.54 (0.036)** | **0.77 (0.04)** |  | **0.54 (0.034)** | **0.78 (0.05)** |
|  |  | **Familial relatives** | **0.51 (0.027)** | **0.78 (0.05)** |  | **0.51 (0.03)** | **0.8 (0.03)** |
|  |  | **sporadic relatives** | **0.52 (0.027)** | **0.78 (0.03)** |  | **0.52 (0.031)** | **0.8 (0.03)** |
|  | **ILF** | **control** | **0.47 (0.039)** | **0.84 (0.05)** |  | **0.47 (0.038)** | **0.84 (0.04)** |
|  |  | **Familial relatives** | **0.46 (0.036)** | **0.85 (0.02)** |  | **0.47 (0.039** | **0.84 (0.02)** |
|  |  | **sporadic relatives** | **0.45 (0.068)** | **0.87 (0.08)** |  | **0.47 (0.041)** | **0.85 (0.04)** |
|  | **SLFP** | **control** | **0.44 (0.033)** | **0.78 (0.04)** |  | **0.44 (0.03)** | **0.79 (0.04)** |
|  |  | **Familial relatives** | **0.45 (0.026)** | **0.78 (0.02)** |  | **0.44 (0.027)** | **0.78 (0.03)** |
|  |  | **sporadic relatives** | **0.44 (0.027)** | **0.79 (0.03)** |  | **0.43 (0.027)** | **0.8 (0.04)** |
|  | **SLFT** | **control** | **0.46 (0.03)** | **0.79 (0.04)** |  | **0.44 (0.033)** | **0.79 (0.03)** |
|  |  | **Familial relatives** | **0.47 (0.03)** | **0.79 (0.03)** |  | **0.44 (0.027)** | **0.78 (0.03)** |
|  |  | **sporadic relatives** | **0.45 (0.028)** | **0.8 (0.03)** |  | **0.43 (0.023)** | **0.79 (0.03)** |
|  | **UNC** | **control** | **0.42 (0.038)** | **0.82 (0.04)** |  | **0.42 (0.034)** | **0.83 (0.03)** |
|  |  | **Familial relatives** | **0.42 (0.04)** | **0.82 (0.03)** |  | **0.42 (0.024)** | **0.83 (0.03)** |
|  |  | **sporadic relatives** | **0.4 (0.035)** | **0.83 (0.03)** |  | **0.41 (0.035)** | **0.83 (0.04)** |

| **(B)** |  | **FA** | **MD e^-3^** |
| --- | --- | --- | --- |
| **Tracts** | **Groups** | **Mean (std)** | |
| **fmajor** | **control** | **0.56 (0.031)** | **0.85 (0.04)** |
|  | **Familial relatives** | **0.56 (0.028)** | **0.87 (0.020** |
|  | **sporadic relatives** | **0.56 (0.041)** | **0.88 (0.04)** |
| **fminor** | **control** | **0.46 (0.056)** | **0.83 (0.04)** |
|  | **Familial relatives** | **0.46 (0.044)** | **0.82 (0.04)** |
|  | **sporadic relatives** | **0.44 (0.042)** | **0.83 (0.04)** |
